# Gallbladder cancer concomitant with autosomal dominant polycystic kidney disease: A case report

**DOI:** 10.1002/ccr3.6734

**Published:** 2022-12-15

**Authors:** Hisashi Murakami, Satoshi Okubo, Masahiro Kobayashi, Miho Akabane, Masaru Matsumura, Junichi Shindoh, Masaji Hashimoto

**Affiliations:** ^1^ Department of Gastroenterological Surgery Toranomon Hospital Tokyo Japan

**Keywords:** ADPKD, gallbladder cancer, TACE

## Abstract

The case is a 67‐year‐old female with autosomal dominant polycystic kidney disease who was followed up regularly. CT scan showed a mural nodule growing over the past 4 years inside the hypodense region surrounded by hepatic cysts. Surgery was performed and the pathological diagnosis was StageI gallbladder cancer.

## Abstract 2

The prognosis of ADPKD has been prolonged, accordingly the number of patients with malignant tumors is expected to increase. There are few reports on the association between ADPKD and gallbladder cancer, and further accumulation of data will be needed.

## 1 INTRODUCTION

Autosomal dominant polycystic kidney disease (ADPKD) is a genetic disorder that occurs in one out of 500–1000 people and is characterized by bilateral progressive renal cysts. Approximately 70% of patients develop end‐stage renal failure at a median age of 58 years.^1^ In addition to renal cysts, ADPKD is also associated with polycystic liver disease, pancreatic duct dilatation, bile duct dilatation, colonic diverticulum, cardiac valve abnormalities, and intracranial aneurysms.^2^ The incidence of hepatic cysts is reported to be approximately 75% in patients aged over 60 years.^3^ Although renal failure used to be the main prognostic factor for ADPKD, prognosis has improved with advances in dialysis therapy. Hepatic complications such as hepatic cyst infection have become the main cause of death.^4^ The association between ADPKD and cancer is under controversial, and there have been no reports of ADPKD with gallbladder cancer. We herein report the case of a 67‐year‐old woman with ADPKD concomitant with gallbladder cancer that was difficult to diagnose.

## 2 CASE REPORT

**FIGURE 1:**
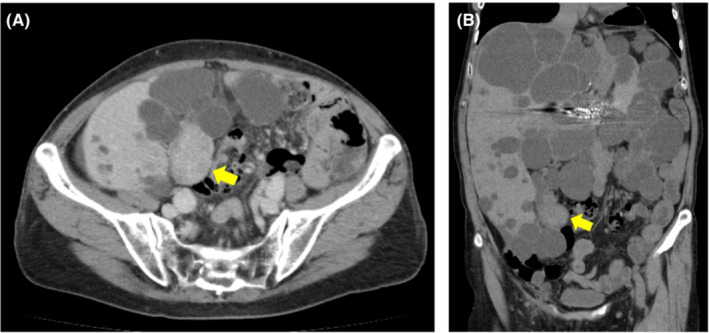
(A, B) Abdominal computed tomography scan. The yellow arrow reveals a 40‐mm‐large hyper dense mass inside a hypodense structure that is seemingly a cyst.

**FIGURE 2:**
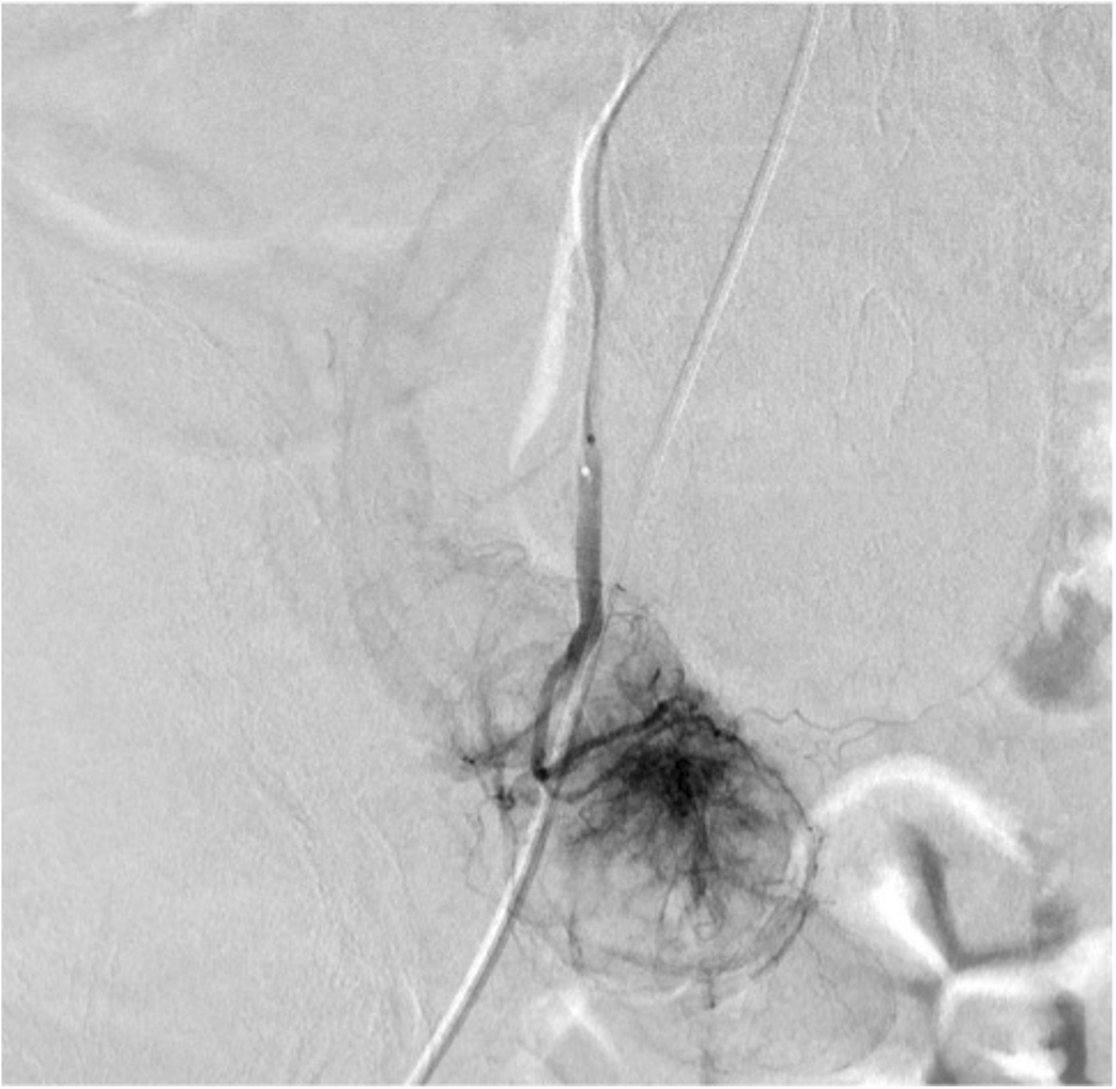
Angiography showed a well‐defined hypervascular stain of 40 mm in size is seen at the tip of the feeding vessel. The feeding artery bifurcated from common hepatic artery just before the bifurcation of proper hepatic artery. Two doses of 30 mg Miriplatin and 1.5 ml of Lipiodol were administered from the main trunk of the feeding vessel. The procedure was completed after confirming Lipiodol deposition by computed tomography.

**FIGURE 3:**
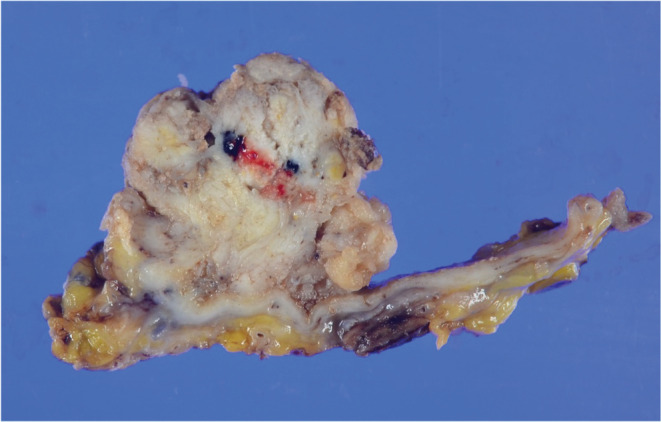
A 40 × 30 × 30‐mm large substantial lesion protruding into the lumen, mainly in the base of the gallbladder

**FIGURE 4:**
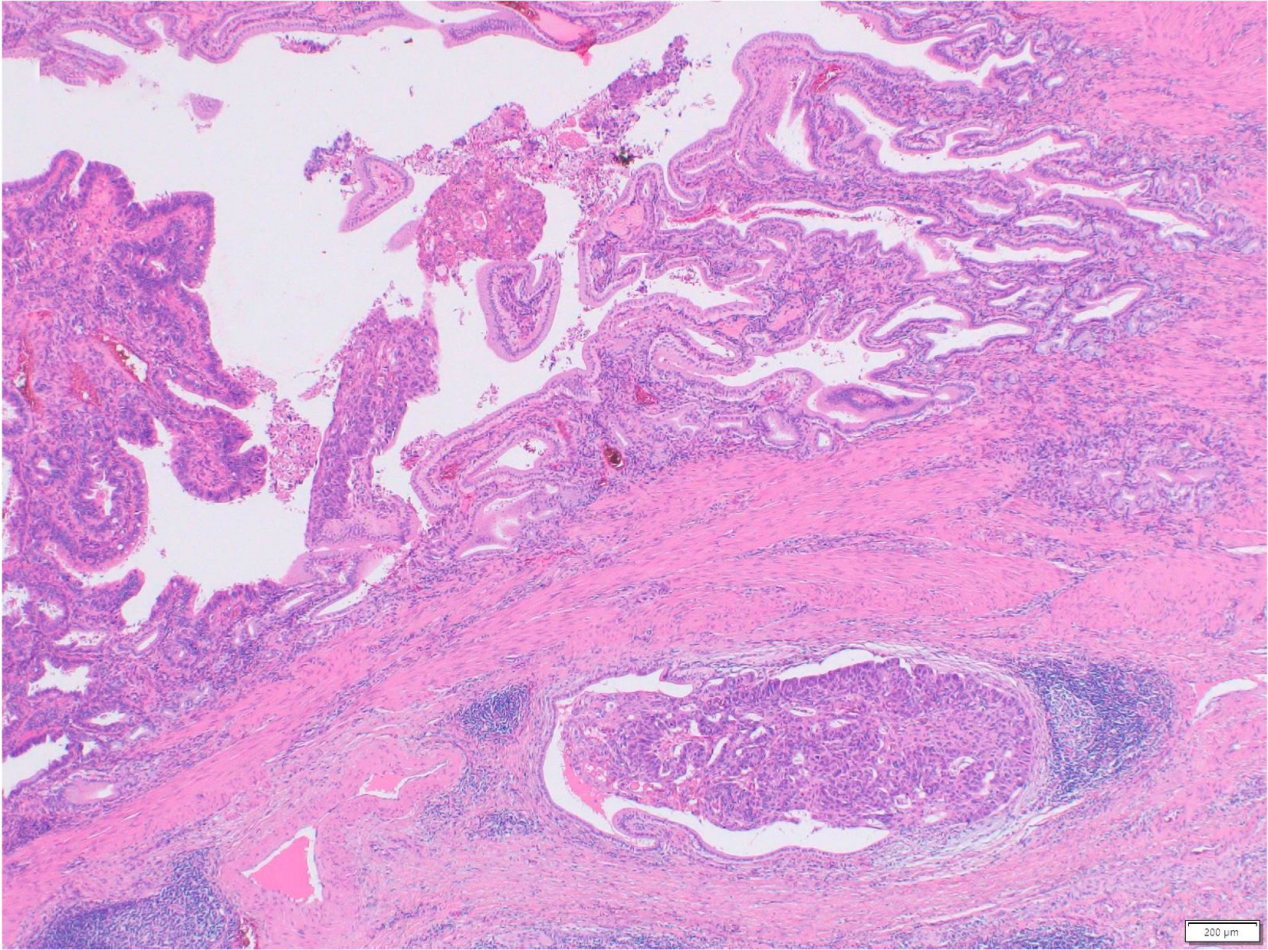
Tumor cells mainly consist of moderately differentiated components. Although some extension into the sub‐serosal layer is observed in the Rokitansky‐Aschoff sinus (RAS), other areas show local extension that remained in the mucosa.

Transcatheter arterial embolization of the left hepatic artery was performed in 2017 for symptoms of abdominal distension associated with multiple hepatic cysts. The patient was followed up regularly in our renal center. A computed tomography (CT) scan of the liver in 2019 showed a mural nodule inside the hypodense region (Figure 1A,B). Hepatic cystadenocarcinoma was suspected because the nodule was shown to gradually increase in size over 2 years from the time of the first visit to our hospital in 2017. Resection was considered for pathological diagnosis, but transcatheter arterial chemoembolization (TACE) was planned considering the surgical risk of the primary disease. Angiographic findings from the common hepatic artery showed a 40‐mm hypervascular stain. Two doses of 30 mg of Miriplatin and 1.5 ml of Lipiodol were administered from the main trunk of the feeding vessel to the tumor (Figure 2). A CT scan 5 months postoperatively showed that the tumor had shrunk, but a CT scan 15 months later showed that it had re‐increased in size. Positron emission tomography CT performed after the re‐increase showed fluorodeoxyglucose accumulation in the mass with a maximum standardized uptake value of 15.7. Moreover, surgical resection was planned in 2021 due to possible tumor regression. Since the tumor was located in the lower abdomen due to multiple hepatic cysts, the abdomen was opened through a midline incision in the lower abdomen. Observation of the abdominal cavity revealed a high degree of adhesion to the greater omentum around the hepatic cysts. After the cyst‐like structure was confirmed to contain the tumor using an echo and detach from the surrounding adhesions, the tumor was found to be in the gallbladder, not in the cyst. It was difficult to identify where the feeding artery came from and the detail structure surrounding tumor because of multiple liver cysts. Cholecystectomy was performed as gallbladder cancer was suspected. The operation time was 63 min with minimal bleeding. The gross finding of the resected specimen revealed a large substantial lesion of 40 × 30 × 30‐mm protruding into the lumen, mainly in the base of the gallbladder (Figure 3). Pathological findings showed a moderately to well‐differentiated component with papillary growth and a poorly differentiated component, as well as strongly atypical tumor cells (Figure 4). The pathological diagnosis was pT1a(M)‐RAS(SS) N0M0 Stage I gallbladder cancer. The patient was discharged from the hospital on the fifth day postoperatively without any apparent complications and was under outpatient observation without recurrence for 1 year postoperatively.

## 3 DISCUSSION

ADPKD is mainly caused by mutations of polycystic kidney disease 1 (PKD1) and polycystic kidney disease 2 (PKD2), which encode for polcystin‐1 (PC‐1) and polycystin‐2 (PC‐2), respectively. PC‐1 and PC‐2 form a complex on the plasma membrane and function as a calcium channel involving in various pathological pathways, including the cAMP and the mTOR pathways.^1^ The former and latter mutations are found in 85% and 15% of cases of ADPKD, respectively. PC‐1 or PC‐2 disruption induces a decrease in intracellular calcium concentration and an increase in cAMP, which leads to abnormal cell proliferation and is associated with the formation of renal and hepatic cysts.^5^

Hepatic cysts are one of the most frequent complications of ADPKD, with a reported incidence of approximately 75% in patients aged over 60 years.^4^ In cases of multiple hepatic cysts, hepatic resection is often associated with postoperative complications due to the anatomical deviation of normal blood vessels and bile ducts.^6^ In this case, we decided to treat the patient with TACE preoperatively considering the higher risk of surgery and the possibility of hepatic cystadenocarcinoma.

The reason behind the difficulty in diagnosing gallbladder cancer was the anatomical deviation of gallbladder due to multiple hepatic cysts. Magnetic resonance cholangiopancreatography (MRCP) and drip infusion cholecystocholangiography CT (DIC‐CT) are generally used to evaluate the travel and anatomy of the bile and gallbladder ducts. However, it was difficult to distinguish the cyst from the gallbladder, even though MRCP was performed before treatment in this case. DIC‐CT can show bile ducts in more detail and may have been useful in recognizing the anatomy of the gallbladder.^7^ Another cause for the difficulty in diagnosis was the absence of cholecystitis after TACE. Generally, the embolization of the gallbladder artery by TACE causes gangrenous cholecystitis due to the obstruction of blood flow to the gallbladder. However, in this case, no cholecystitis occurred despite performing TACE on the gallbladder artery, which was the feeding vessel for the tumor. It has been reported that PKD1 and PKD2 produce inflammatory cells in the early stages of the disease, inducing severe adhesions to the surrounding tissues.^8^ It was inferred from intraoperative findings that the gallbladder did not become ischemic due to the blood flow from the greater omentum, which was associated with the aforementioned severe adhesions.

Gallbladder carcinoma is considered to be a tumor with a poor prognosis due to the lack of symptoms and the tendency to invade the gallbladder bed, where the serosal layer is deficient.^9^ The overall mean survival rate of patients with gallbladder cancer is reportedly 19 months, with a 5‐year survival rate of 28.8%.^10^ However, in this case, the depth of the lesion was limited to the mucosa, although the time since onset to operation was 4 years. The cause of the slow progression in this case is unclear, but two possibilities are suggested. One is that the disease may have been suppressed by TACE. Tumor shrinkage was observed 5 months after TACE, indicating its effectiveness. However, tumor regression was observed 15 months later, and postoperative pathology results showed little necrosis or other changes caused by TACE, suggesting that it is unclear whether TACE directly contributed to cancer progression. The other cause is considered to be due to the PKD1 gene. In vitro experiments have shown that the PKD1 gene promotes cell adhesion and attenuates metastasis and the invasion of tumor cells,^11^ suggesting that it may have been involved in tumor suppression. However, few reports have shown an association between ADPKD and cancer, and the relevance is still controversial. Moreover, there are no reports on gallbladder cancer in patients with ADKPD; further research with long‐term prognoses is warranted.

We experienced a case of gallbladder cancer associated with ADPKD that was difficult to diagnose because of the anatomical deviation of the gallbladder and the absence of cholecystitis after TACE. ADPKD is now expected to have a long‐term prognosis. Therefore, it is important to perform periodic imaging examinations all while taking into consideration malignant complications.

## AUTHOR CONTRIBUTIONS

**Hisashi Murakami:** Conceptualization; writing – original draft; writing – review and editing. **Satoshi Okubo:** Supervision; writing – review and editing. **Masahiro Kobayashi:** Writing – review and editing. **Miho Akabane:** Writing – review and editing. **Masaru Matsumura:** Writing – review and editing. **Junichi Shindoh:** Writing – review and editing. **Masaji Hashimoto:** Writing – review and editing.

## CONFLICT OF INTEREST

The authors have no conflicts of interest directly relevant to the content of this article.

## STATEMENT OF HUMAN AND ANIMAL RIGHTS

All methods were performed in accordance with the relevant guidelines and regulations including the Declaration of Helsinki.

## CONSENT

Formal consent is not required for this type of study.

## Data Availability

All authors are requested to make sure that all data and materials as well as software application or custom code support their published claims and comply with field standards. Please note that journals may have individual policies on (sharing) research data in concordance with disciplinary norms and expectations. The data that support the findings of this study are available on request from the corresponding author, HM.

## References

[1] Rastogi A , Ameen KM , Al‐Baghdadi M , et al. Autosomal dominant polycystic kidney disease: updated perspectives. Ther Clin Risk Manag. 2019;15:1041‐1052. 31692482 10.2147/TCRM.S196244 PMC6716585

[2] Sun K , Dechao X , Mei C . The association between autosomal dominant polycystic kidney disease and cancer. Int Urol Nephrol. 2019;51:93‐100. 30109558 10.1007/s11255-018-1951-5

[3] Milutinovic J , Fialkow PJ , Rudd TG , Agodoa LY , Phillips LA , Bryant JI . Liver cysts in patients with autosomal dominant polycystic kidney disease. Am J Med. 1980;68:741‐744. 7377224 10.1016/0002-9343(80)90266-1

[4] Fick GM , Johnson AM , Hammond WS , Gabow PA . Causes of death in autosomal dominant polycystic kidney disease. J Am Soc Nephrol. 1995;5:2048‐2056. 7579053 10.1681/ASN.V5122048

[5] Kim DY , Park JH . Genetic mechanisms of ADPKD. Adv Exp Med Biol. 2016;933:13‐22. 27730431 10.1007/978-981-10-2041-4_2

[6] Martinez‐Perez A , Alberola‐Soler A , Domingo‐Del Pozo C , Pemartin‐Comella B , Martinez‐Lopez E , Vazquez‐Tarragon A . Laparoscopic surgery and polycystic liver disea se: Clinicopathological features and new trends in management. J Minim Access Surg. 2016;12:265‐70. 27279400 10.4103/0972-9941.169976 PMC4916755

[7] Ishii H , Noguchi A , Fukami T , et al. Preoperative evaluation of accessory hepatic ducts by drip infusion cholangiography with CT. BMC Surg. 2017;17:52. 28482819 10.1186/s12893-017-0251-9 PMC5422935

[8] Mangos S , Lam PY , Zhao A , et al. The ADPKD genes pkd1a/b and pkd2 regulate extracellular matrix formation. Dis Model Mech. 2010;3:354‐365. 20335443 10.1242/dmm.003194 PMC2860853

[9] Sharma A , Sharma KL , Gupta A , Yadav A , Kumar A . Gallbladder cancer epidemiology, pathogenesis and molecular genetics: Recent update. World J Gastroenterol. 2017;23:3978‐3998. 28652652 10.3748/wjg.v23.i22.3978 PMC5473118

[10] Zhu X , Zhang X , Hu X , et al. Survival analysis of patients with primary gallbladder cancer from 2010 to 2015: A retrospective study based on SEER data. Medicine (Baltimore). 2020;99:e22292 33019404 10.1097/MD.0000000000022292 PMC7535694

[11] Zhang K , Ye C , Zhou Q , et al. PKD1 inhibits cancer cells migration and invasion via Wnt signaling pathway in vitro. Cell Biochem Funct. 2007;25:767‐74. 17437318 10.1002/cbf.1417

